# A 29‐year‐old man with a right intracranial mass

**DOI:** 10.1111/bpa.13143

**Published:** 2023-01-30

**Authors:** Leiming Wang, Yukui Wei, Lianghong Teng

**Affiliations:** ^1^ Department of Pathology Xuanwu Hospital, Capital Medical University Beijing China; ^2^ National Center for Neurological Disorders Beijing China; ^3^ Department of Neurosurgery Xuanwu Hospital, Capital Medical University Beijing China

**Keywords:** adult, *DICER1*, primary intracranial sarcoma

## 1 CLINICAL HISTORY AND IMAGING

**FIGURE 1:**
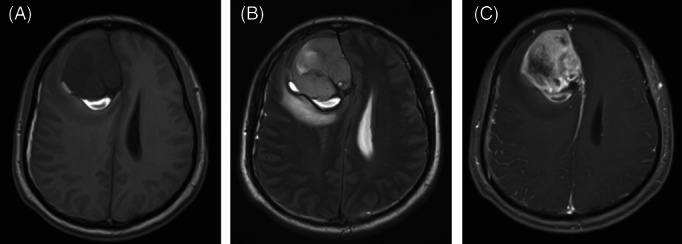
Magnetic resonance imaging showed a mass located in the right frontal region with heterogeneous isointense T2‐weighted (A) and hypointense T1‐weighted (B) signal. After gadolinium administration, the mass showed heterogeneous enhancement (C).

A 29‐year‐old man presented with intermittent headache for 3 months. Cranial magnetic resonance imaging showed a nodular, hemorrhagic mass measuring 57 × 56 × 51 mm located in the right frontal region. The mass showed heterogeneously isointense T2‐weighted and hypointense T1‐weighted image signals with heterogeneous contrast enhancement using gadolinium (Figure 1). The patient underwent systemic examinations during which no other specific abnormalities were found. Gross total resection was performed by a right frontal approach. During the operation, the right frontal gyrus was found to be swollen. The tumor was located below the right frontal cortex, and adhered tightly to the cerebral falx (Box 1).

BOX 1 **Virtual glass slide**

Access at https://isn‐slidearchive.org/?col=ISN&fol=Archive&file=BPA‐22‐10‐243.svs

## 2 FINDINGS

**FIGURE 2:**
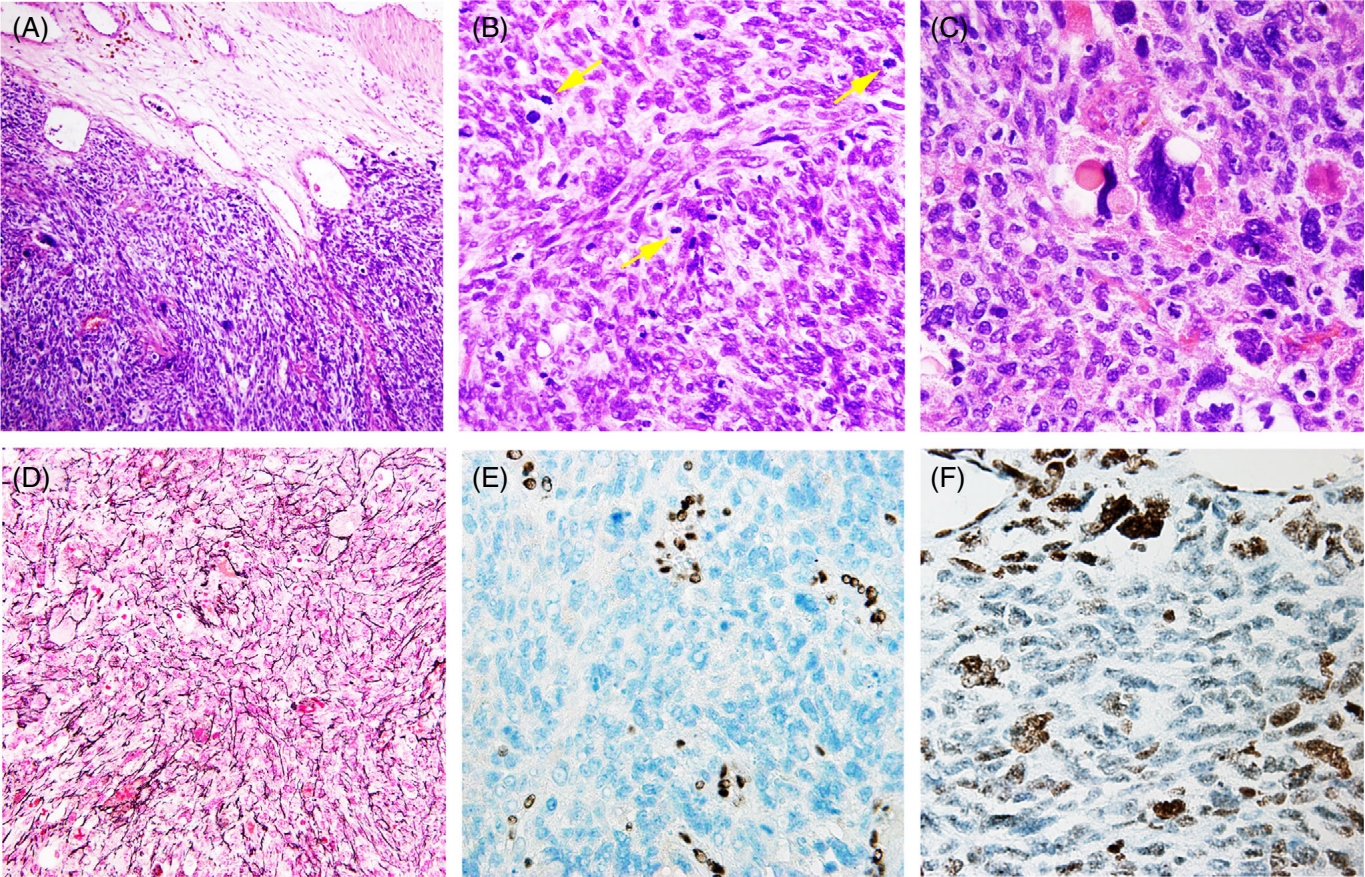
(A) Hematoxylin and eosin staining showed a densely cellular malignant tumor involving meninges. (B) Mitoses were frequent (arrow). (C) some of the tumor cells had prominent eosinophilic cytoplasmic globules. (D) Reticulin staining revealed abundant reticulin network deposition between tumor cells. (E) Immunoreactive for ATRX. (F) Immunoreactive for H3K27me3. Magnification ×100 (A), ×400 (D), ×400 (B, C, E, F).

Histopathological examination revealed a densely cellular malignant tumor involving meninges (Figure 2A). The tumor exhibited a poorly differentiated and somewhat primitive appearance. There was a heterogeneous population of small round cells, spindle cells and ovoid cells arranged in fascicular and storiform patterns. The tumor cells exhibited obvious atypia, with scattered pleomorphic cells. Mitoses were frequent (10 mitoses/mm^2^) (Figure 2B). Some of the tumor cells had prominent eosinophilic cytoplasmic globules (Figure 2C). Reticulin stain revealed a dense pericellular network among tumor cells (Figure 2D). By immunohistochemistry, tumor cells were diffusely positive for vimentin and negative for GFAP, OLIG2 and synaptophysin. They expressed diffusely p53 and showed loss of expression for ATRX (Figure 2E) and H3 K27me3 (mosaic loss) (Figure 2F). INI1 protein expression was retained, whereas H3G34R, H3G34V, EMA, SSTR‐2, CD34, STAT‐6, SOX‐10, SMA, Desmin and Myogenin were negative. The Ki‐67 proliferation index was 80%. Next‐generation sequencing revealed that the tumor harbored mutations of *DICER1* (c.5438A>G, p.E1813G mutation and c.4458dup, p.Ser1487IIefsTer5 mutation, with mutant allele frequencies of 45.1% and 49.3% respectively), while *H3.1*/*H3.3* and *IDH1*/*2* were wild‐type. In addition, the tumor also harbored *NF1* nonsense mutation (c.6211C>T p.Gln2071Ter p.Q2071*), *ARID1B* missense mutation (c.6565C>G p.Pro2189Ala), *ATRX* splicing mutation (c.6505‐1G>C), and *TP53* nonsense mutation (c.517G>A p.Val173Met p.V173M), as well as copy gain of *FGFR3*, *NOTCH1*, and *SDHA*.

## 3 DIAGNOSIS

Primary intracranial sarcoma, *DICER1*‐mutant.

## 4 DISCUSSION

*DICER1*‐mutant primary intracranial sarcoma, a very rare tumor recently added to the 2021 WHO classification of central nervous system (CNS) tumors, harbors pathogenic *DICER1* mutations and a distinctive DNA‐methylation profile [1]. The *DICER1* gene, located at 14q32.2, encodes a ribonuclease essential in the production of microRNAs and its mutations have been shown to interfere with its ability to process RNAs. The tumor primarily occurs in children, with a median age of 6 years and has been associated primarily with *DICER1* tumor predisposition syndrome, but also with neurofibromatosis type 1 [1]. The prognosis remains unknown because of limited clinical data collected to date.

Here, we report a very unusual case occurring in an adult, with no indications of *DICER1* tumor predisposition syndrome. This tumor was initially considered a possible H3 G34‐mutant diffuse hemispheric glioma or embryonal CNS tumor according to histological features indicative of a malignant high grade tumor with a somewhat primitive appearance, negative for OLIG2, with loss of ATRX expression and strong p53 expression [2]. Given the complete lack of expression of glial (GFAP and OLIG2) markers, while vimentin was diffusely positive in the tumor cells and reticulin stain showed a dense pericellular network, consideration was also given to other diagnoses including malignant peripheral nerve sheath tumor, solitary fibrous tumor or an undifferentiated sarcoma. The final diagnosis of “*DICER1*‐mutant primary intracranial sarcoma” was made after confirming that this tumor harbored *DICER1* mutations. In addition to *DICER1* mutations, the tumor also showed disruptions of p53 signaling due to a TP53 mutation, chromatin remodeling due to a ATRX mutation, and NOTCH signaling due to a NOTCH1 mutation, as well as the activation of the MAPK signaling pathway due to alterations in NF1 and FGFR3, which are frequent in *DICER1*‐mutant primary intracranial sarcoma [1, 3].

The exact histogenesis of primary intracranial sarcoma, *DICER1*‐mutant is at present unknown. Its relationship to *DICER1*‐associated sarcoma occurring at extracranial anatomical sites including lung, uterus, kidney, and others also remains to be determined.

Our patient received radiotherapy after surgery, but unfortunately suffered from tumor recurrence after 3 months and died after 9 months.

## AUTHOR CONTRIBUTION

Leiming Wang analyzed the data, reviewed the pathological diagnosis and wrote the manuscript. Yukui Wei provided essential material and analyzed the clinical data. Lianghong Teng reviewed the pathological diagnosis and the manuscript. All authors approved the final version of the manuscript.

## CONFLICT OF INTEREST

The authors declare no conflict of interest.

## ETHICS STATEMENT

The study was approved by the ethics committee of Xuanwu Hospital, Capital Medical University, Beijing, China, and was conducted in full compliance with all principles of the Helsinki Declaration.

## Data Availability

I confirm that my article contains a Data Availability Statement even if no data is available (list of sample statements) unless my article type does not require one (e.g., Editorials, Corrections, Book Reviews, etc.).

## References

[1] WHO Classification of Tumours Editorial Board . Central nervous sytem. International Agency for Research on Cancer. 2021. https://tumourclassificationiarc.who.int/chapters/8

[2] Wang L , Shao L , Li H , Yao K , Duan Z , Zhi C , et al. Histone H3.3 G34‐mutant diffuse gliomas in adults. Am J Surg Pathol. 2022;46:249–57. 34352809 10.1097/PAS.0000000000001781

[3] McCluggage WG , Foulkes WD . *DICER1*‐associated sarcomas: towards a unified nomenclature. Mod Pathol. 2021;34:1226–8. 32572152 10.1038/s41379-020-0602-4

